# Stability of selected reference genes in Sf9 cells treated with extrinsic apoptotic agents

**DOI:** 10.1038/s41598-019-50667-2

**Published:** 2019-10-02

**Authors:** Benshui Shu, Jingjing Zhang, Jie Zeng, Gaofeng Cui, Guohua Zhong

**Affiliations:** 1 Guangzhou City Key Laboratory of Subtropical Fruit Trees Outbreak Control, Zhongkai University of Agriculture and Engineering, Guangzhou, China; 2 Key Laboratory of Crop Integrated Pest Management in South China, Ministry of Agriculture and Rural Affairs, South China Agricultural University, Guangzhou, China; 3 Key Laboratory of Natural Pesticide and Chemical Biology, Ministry of Education, South China Agricultural University, Guangzhou, China

**Keywords:** Apoptosis, Entomology

## Abstract

As a tightly controlled cell death process, apoptosis eliminates unwanted cells and plays a vital role in multicellular organisms. Previous study have demonstrated that apoptosis occurred in *Spodoptera frugiperda* cultured Sf9 cells, which triggered by diverse apoptotic stimuli, including azadirachtin, camptothecin and ultraviolet. Due to its simplicity, high sensitivity and reliable specificity, RT-qPCR has been used widespread for analyzing expression levels of target genes. However, the selection of reference genes influences the accuracy of results profoundly. In this study, eight genes were selected for analyses of their suitability as references for normalizing RT-PCR data in Sf9 cells treated with apoptotic agents. Five algorithms, including NormFinder, BestKeeper, Delta Ct method, geNorm, and RefFinder, were used for stability ranking. Based on comprehensively analysis, the expression stability of selected genes varied in cells with different apoptotic stimuli. The best choices for cells under different apoptosis conditions were listed: EF2 and EF1α for cells treated with azadirachtin; RPL13 and RPL3 for cells treated with camptothecin; EF1α and β-1-TUB for cells irradiated under ultraviolet; and EF1α and EF2 for combinational analyses of samples. Our results not only facilitate a more accurate normalization for RT-qPCR data, but also provide the reliable assurance for further studies of apoptotic mechanisms under different stimulus in Sf9 cells.

## Introduction

The insect cell line Sf9 derived from fall armyworm moth *Spodoptera frugiperda* was widely used for biotechnological applications in both academic laboratories and industry^1,2^. It is one of the common cell lines used for recombinant protein expression using the baculovirus-insect cell expression system due to its superiority of higher protein yields than that of mammalian cell lines and *Xenopus laevis* oocytes expression systems^3,4^. Besides, it is also considered as a stable and comparable standardized evaluation model for insecticide cytotoxicology^5^. Recently, Sf9 cells have also been used for research on apoptosis. Many substances, such as azadirachtin, abamectin, camptothecin, spinosad and ultraviolet, could induce apoptosis in Sf9 cells^6–9^.

Apoptosis is a normal physiological process for elimination of unwanted cells and works as a homeostatic mechanism in the development of multicellular organisms, and as the defense mechanism in response to a wide variety of stimuli^10,11^. It is an irreversible process and is tightly mediated by a set of core genes, such as p53, caspases and IAP antagonists^12–14^. Similar to studies on apoptosis in the silkworm *Bombyx mori*, studies on apoptosis in Sf9 cells focus mainly on two aspects, morphological changes after apoptotic stimulation and analysis of core apoptosis-related genes^15^. With the continuous advances in sequencing technology, the molecular mechanism of apoptosis in Sf9 cells is expected to be further studied.

A series of detection methods could be used to detect apoptosis, including changes in morphology, occurrence of DNA ladder and TUNEL. At the same time, changes in transcription levels of apoptosis-related genes are other important indicators for apoptosis. For example, the expression levels of RHG family members including reaper, hid, grim in organisms foreshadow the occurrence of apoptosis^14^. The independent transcription of tumor suppressor p53 regulates mitochondrial membrane permeabilization and apoptosis by enhancing the transcriptional expression of a large number of genes involved in apoptosis, including Bax^16^. Forkhead box transcription factor (FoxO) mediated extrinsic apoptotic pathway through activating the transcription levels of FasL and TRAIL, and eventually induced apoptosis^17^.

Real-time reverse transcription polymerase chain reaction (RT-qPCR) is considered as the gold standard for quantifying mRNA levels of target genes^18,19^. Due to the advantages of simple operation, easy to analysis, high sensitivity and reliable specificity, this technique has been widely used in various fields, such as basic research, molecular medicine and biotechnology. However, it also engenders some new aspects that need to pay attention^20^. For example, the factors including RNA quality and efficiency of both reverse transcription and primer extension may interfere with the accuracy of RT-qPCR results^18^. Therefore, reference genes with stable expression under different situations are included to reduce potential inaccuracy^21^. Usually genes encoding cytoskeletal proteins, GAPDH, ribosomal proteins are thought with stable expression, and therefore are often chosen for standardization in RT-qPCR experiments^22^. However, more and more evidence suggests that these assumed stable genes are actually strongly influenced by various conditions, especially apoptosis. For example, elevated expression of GAPDH is found in cells undergoing apoptosis^23,24^. Actin and tubulin are disturbed in apoptotic cells induced by azadirachtin^25,26^. In order to get the more accurate results, candidates for reference genes should be determined under specific experimental conditions before performing qRT-PCR analyses.

In this study, eight genes that have been routinely used as reference genes in previous studies were chosen for detailed analyses in Sf9 cells. The selected genes are those encoding Actin, elongation factor 1 alpha (EF1α), elongation factor 2 (EF-2), glyceraldehyde-3-phosphate dehydrogenase (GAPDH), ribosomal protein L3 (RPL3), ribosomal protein L13 (RPL13), alpha-tubulin (α-TUB), and beta-1-tubulin (β-1-TUB). Moreover, the expression stability of selected genes under apoptotic conditions in Sf9 cells were analyzed by NormFinder, BestKeeper, Delta Ct method, geNorm, and RefFinder. Our results represent the right combination of reference genes for qRT-PCR experiments in Sf9 cells under apoptosis conditions, which could provide useful information for future analyses of gene expression related to apoptosis mechanism.

## Results

### Amplification efficiency and expression profiling of candidate reference genes

**Figure 1:**
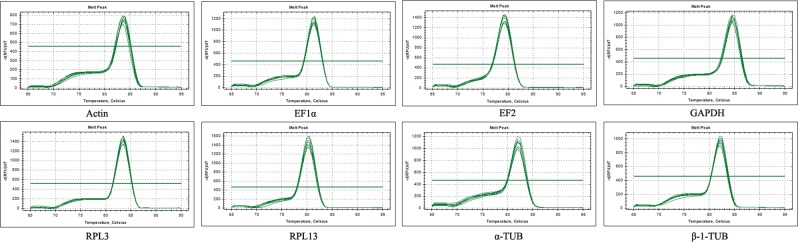
Melting curves of eight candidate reference genes. Eight candidate reference genes were showed as Actin, EF1α, EF2, GAPDH, RPL3, RPL13, α-TUB and β-1-TUB.

According to the sequences of eight candidate reference genes, the primer pairs for each reference gene were designed to produce 80–250 bp PCR fragments. Primer specificity for each gene was validated by a melting curve analysis in qRT-PCR and a single peak was observed in each case (Fig. 1). In addition, amplification efficiencies ranged from 92.2% to 107% based on standard curves for eight genes, and correlation coefficients (R^2^) were greater than 0.99, which fit the requirements of RT-qPCR (Table 1).

**Table 1:** Details of the primer pairs used for real-time PCR of 8 housekeeping genes of Sf9 cells.

Gene name	GenBank accession number	Primer sequenses	Product length (bp)	Efficiency (%)	R^2^	Slope
RPL13	AF400183.1	F: 5′ GCCTTAACCCTGCTTTTGCTAG 3′	160	92.2	1	−3.523
		R: 5′ GCTTCGCCCTTCAATACCTTC 3′				
GAPDH	KC262638.1	F: 5′ ACTGTTGACGGACCCTCTGGAA 3′	152	96.3	0.999	−3.413
		R: 5′ ACGGGAACACGGAAAGCCAT 3′				
EF1α	U20139.1	F: 5′ TGGGCGTCAACAAAATGGA 3′	148	96.1	0.999	−3.419
		R: 5′ TCTCCGTGCCAGCCAGAAAT 3′				
α−TUB	HQ008728.1	F: 5′ TCGGCAAGGAAATCGTAGACC 3′	96	98.1	0.998	−3.368
		R: 5′ CGAAGGAGTGGAAGATAAGGAAGC 3′				
β-1-TUB	AF548017.1	F: 5′ TCAGGCGCAAGGCTTTCTT 3′	97	107	0.992	−3.624
		R: 5′ TCGGACACCAGGTCGTTCAT 3′				
Actin	HQ008727.1	F: 5′ TCCCCATCTACGAAGGTTACGC 3′	124	94.7	0.998	−3.455
		R: 5′ GCGGTGGTGGTGAAAGAGTAAC 3′				
EF2	/	F: 5′ AGCGTGAGAAGAGTGAAAAGGG 3′	116	94.5	0.999	−3.451
		R: 5′ GACCACAAGAGCACCATCAGTTA 3′				
RPL3	AY072287.1	F: 5′ AAGCCAGTCCACCTTACCGC 3′	206	96.4	0.995	−3.41
		R: 5′ GCCCAAACAGTGAGCAGAGC 3′				

**Figure 2:**
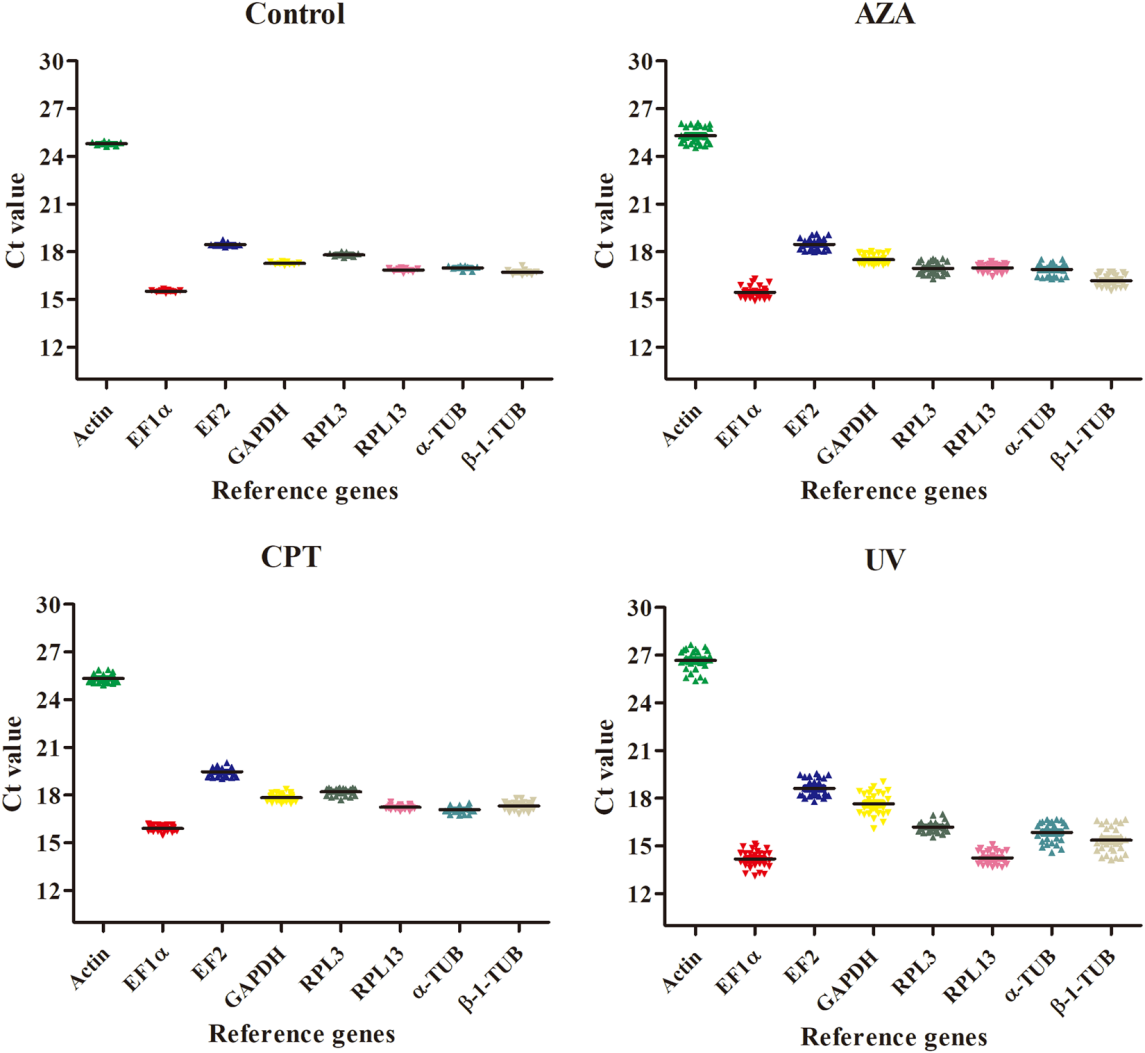
Expression profiles of eight candidate reference genes in Sf9 cells under different conditions.

The raw CT values of reference genes in different samples varied significantly and ranged from 14.92 to 28.24. EF1α exhibited the smallest CT value (average 16.4 ± 1.20), but had the highest expression level. To the opposite, actin exhibited the largest CT value (25.92 ± 1.13), but had the lowest expression level. Besides, the mean CT values of remaining reference genes including EF2, GAPDH, RPL3, RPL13, α-Tubulin and β-1-Tubulin were 19.64 ± 1.31, 18.55 ± 1.50, 18.22 ± 1.10, 17.42 ± 0.57, 17.60 ± 1.03 and 17.55 ± 1.34, respectively. Since CT values for all genes were within 15–30, all the genes were considered suitable for further analyses (Fig. 2).

### Stability of reference genes under abiotic conditions

#### Azadirachtin treatment

**Figure 3:**
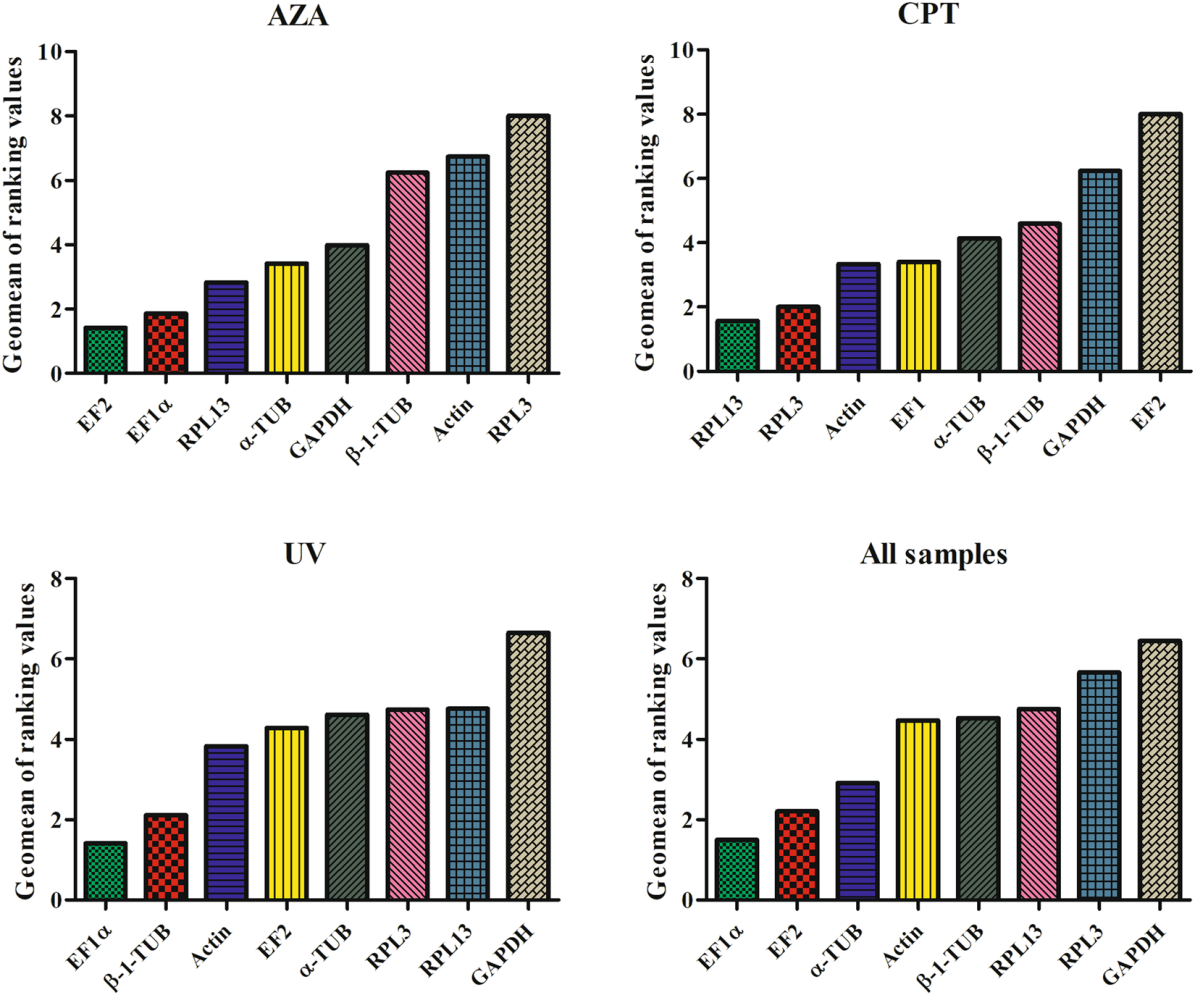
Comprehensive ranking of eight candidate reference genes in Sf9 cells with different treatments analyzed by RefFinder. The reference gene with more stable expression has the lower Geomean of ranking value.

**Figure 4:**
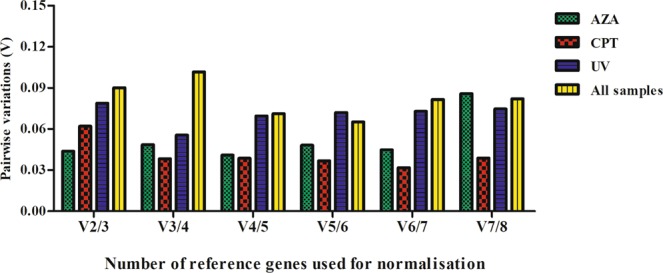
The Pairwise variation values in different experimental conditions according to geNorm. The value of Vn/Vn + 1 < 0.15 means n should be the optimal number of reference gene selection in RT-qPCR analysis.

The rankings for the expression stability of these eight genes in Sf9 cells treated with azadirachtin, camptothecin, and ultraviolet, respectively, were analyzed using different algorithms (Table 2, Fig. 2). For cells treated with azadirachtin, the ranking obtained with Delta CT was exactly the same to that obtained with Normfinder, and slightly different from that obtained with geNorm. EF2, EF1α and α-TUB were shown to be the most stable genes. However, the ranking obtained with BestKeeper was quite different, with RPL13 and GAPDH as the most stable genes. (Table 2). According to the results obtained using RefFinder, the stability of these eight genes in Sf9 cells treated with azadirachtin was in following order: EF2 > EF1α > RPL13 > α-TUB > GAPDH > β-1-TUB > Actin > RPL3 (Fig. 3). In addition, the pairwise variations of V2/3 to V7/8 were less than 0.15 (Fig. 4), which means at least two of the selected genes should be used to obtain the accurate RT-qPCR results. Therefore, EF2 and EF1α were considered to be the suitable combination as reference genes for RT-qPCR analyses in Sf9 cells treated with azadirachtin.

**Table 2:** Expression stability of the eight candidate reference genes in Sf9 cells by different algorithms.

Abiotic conditions	Candidate Reference genes	Delta CT		geNorm		Normfinder		BestKeeper	
		Rank	Stability	Rank	Stability	Rank	Stability	Rank	Stability
Azadirachtin	Actin	7	0.433	6	0.261	7	0.371	7	0.409
	EF1α	2	0.31	1	0.163	2	0.106	3	0.237
	EF2	1	0.299	1	0.163	1	0.087	4	0.239
	GAPDH	5	0.346	5	0.229	5	0.187	2	0.23
	RPL3	8	0.696	8	0.393	8	0.668	8	0.458
	RPL13	4	0.333	4	0.209	4	0.164	1	0.178
	α-TUB	3	0.322	3	0.181	3	0.158	5	0.285
	β-1-TUB	6	0.402	7	0.291	6	0.276	6	0.376
Camptothecin	Actin	5	0.302	1	0.165	5	0.229	5	0.254
	EF1α	3	0.266	5	0.242	3	0.156	3	0.195
	EF2	8	0.363	8	0.292	8	0.314	8	0.357
	GAPDH	6	0.312	7	0.219	6	0.24	6	0.27
	RPL3	2	0.256	1	0.165	2	0.143	4	0.236
	RPL13	1	0.243	3	0.269	1	0.106	2	0.17
	α-TUB	7	0.316	6	0.253	7	0.256	1	0.135
	β-1-TUB	4	0.277	4	0.232	4	0.175	7	0.296
Ultraviolet	Actin	3	0.414	4	0.291	3	0.204	6	1.055
	EF1α	1	0.393	1	0.225	1	0.15	4	0.992
	EF2	4	0.439	3	0.278	4	0.295	7	1.132
	GAPDH	7	0.577	5	0.333	7	0.525	8	1.314
	RPL3	6	0.516	7	0.43	6	0.4	2	0.689
	RPL13	8	0.632	8	0.48	8	0.58	1	0.516
	α-TUB	5	0.471	6	0.382	5	0.305	3	0.865
	β-1-TUB	2	0.402	1	0.225	2	0.184	5	1.022
All Samples	Actin	5	0.514	5	0.405	4	0.329	4	0.96
	EF1α	1	0.433	1	0.285	1	0.145	5	1.04
	EF2	2	0.472	1	0.285	2	0.266	6	1.117
	GAPDH	6	0.592	6	0.431	6	0.483	8	1.311
	RPL3	7	0.642	7	0.489	7	0.52	3	0.904
	RPL13	8	0.722	8	0.547	8	0.642	1	0.47
	α-TUB	3	0.489	4	0.385	3	0.273	2	0.856
	β-1-TUB	4	0.512	3	0.319	5	0.34	7	1.12

#### Camptothecin treatment

Similarly, for cells treated with camptothecin, again the ranking obtained using Delta CT was exactly the same as that obtained with Normfinder (Table 2) and RPL13, RPL3 and EF1α were considered to be the ideal reference genes. Both geNorm and BestKeeper yielded different ranking, with α-TUB, RPL13 and EF1α as the most stable genes from the BestKeeper ranking, whereas RPL3, actin and RPL13 as the most stable genes from the geNorm ranking (Table 2). Based on RefFinder analyses, the stability of these eight genes in Sf9 cells treated with camptothecin was in following order: RPL13 > RPL3 > Actin > EF1α > α-TUB > β-1-TUB > GAPDH > EF2 (Fig. 3). Besides, EF2 was ranked as the least stable gene by all computational programs, and therefore was not recommended as a reference gene for qRT-PCR analyses in cells treated with camptothecin. Additionally, pairwise variation values of V2/3 to V7/8 generated by geNorm were less than 0.15 (Fig. 4). Hence, RPL13 and RPL3 were sufficient as reference genes for normalizing RT-qPCR results in Sf9 cells treated with camptothecin.

#### Ultraviolet treatment

For cells irradiated with ultraviolet, Delta CT and Normfinder yielded the same ranking as well, and with EF1α, β-1-TUB and actin as the most stable genes, whereas GAPDH and RPL13 as the least stable ones. The results of geNorm was similar to the results of Normfinder and EF1α, β-1-TUB was arrayed as the most stable genes. The results of BestKeeper were different from the results of other algorithms which RPL13 and RPL3 were considered to be expressed more stable, and GAPDH was the unstable one (Table 2). RefFinder results showed more similar to Normfinder results, which indicated EF1α, β-1-TUB and Actin were the stable genes, and followed with EF2 and α-TUB (Fig. 3). In addition, GAPDH, RPL13 and RPL3 were considered unstable. The results of geNorm also proposed that two reference genes should be used in cells irradiated with UV. Thus, EF1α and β-1-TUB could be the best choices of reference genes for normalizing RT-qPCR data in ultraviolet-irradiated samples.

#### The stability ranking of reference genes over all treatments

For all the samples under different treatments, EF1α and EF2 were ranked as the most stable in all analysis programs except BestKeeper. In addition, RPL3 and RPL13 were identified as the least stable genes. BestKeeper analysis revealed that RPL13 and α-TUB could be required for normalization (Table 2). The ranking of RefFinder results listed from most stable gene to least stable gene was showed as followed: EF1α > EF2 > α-TUB > Actin > β-1-TUB > RPL13 > RPL3 > GAPDH (Fig. 3). The pairwise variation V2/3 was calculated by geNorm and was below 0.15 (Fig. 4). Therefore, EF1α and EF2 were taken as the best reference genes for cells treated with agents that induce apoptosis.

## Discussion

Azadirachtin, a tetranortriterpenoid compound, is an environmentally friendly botanical insecticide and has a broad spectrum of insecticidal effect, including mortality, antifeeding, repellent, disruption of growth and development^27,28^. Toxicological research demonstrated that apoptosis induction is likely an important mechanism for insecticidal of azadirachtin. Apoptosis has been reported in different insect cell lines treated with azadirachtin, including SL-1 (*S. litura*), BTI-Tn-5B1–4 (*Trichoplusia ni*) and S2 (*Drosophila melanogaster*)^26,29,30^. In addition, it has also been observed in prothoracic glands of *Bombyx mori* and in midgut of *S. litura* after treated with azadirachtin^31,32^. Our previous studies indicated that azadirachtin could activate mitochondrial and lysosomal pathways that lead to apoptosis in Sf9 cells^1,8,33^. In most previous studies, researchers have arbitrarily selected genes as internal references for analyzing the molecular mechanism of azadirachtin-induced apoptosis. However, some reference genes could be involved in apoptosis process, instead of maintaining the functions of cells. Therefore, their expression may be affected as well. Actin is thought a target for azadirachtin action. There is evidence to show that azadirachtin can bind to actin and inhibit its polymerization based on immunohistochemistry and in silico analyses in *D. melanogaster*^25,34^. Additionally, similar phenomenon was also observed in *Plutella*^35^. Furthermore, cytoskeleton has been found to be destroyed by azadirachtin in Sf9 cells due to the impact of azadirachtin on actin and β-tubulin^26,36^. Besides, ribosomal protein L9 was also affected by azadirachtin in Sf9 cells^36^. In this study, the stability of eight reference genes in Sf9 cells with azadirachtin treatments was assessed by five analysis programs. In cells treated with azadirachtin, EF2 and EF1α were considered stable genes, whereas actin, tubulins and RPL3 was considered to be unstable. Our results were consistent with previous reports, which showed that EF2 and EF1α were the most appropriate reference genes for normalizing RT-qPCR data in Sf9 cells treated with azadirachtin.

Camptothecin is an indole alkaloid isolated from *Camptotheca acuminate* used as an important drug for cancer therapy^37^. Currently, the significant insecticidal activities of CPT and its derivatives have been documented^38^. In addition, CPT could induce apoptosis in insect cell lines, including IOZCAS-Spex-II (*Spodoptera exigua*), SL-1 (*S. litura*), Sf21 and Sf9 (*S. frugiperda*)^8,39–42^. Furthermore, the midgut epithelial cell apoptosis was observed in *S. litura* after treated with CPT for one week^43^. At present, the expression levels of reference genes affected by CPT and its derivatives have not been concerned and rarely reported. Expression stability of selected genes has been initially analyzed in human ovarian cancer cell lines UACC-1598 and SKOV3 with a camptothecin derivative, and demonstrated that ribosomal protein L13a (RPL13A) is stable as a reference gene whereas GAPDH is unstable^44^. In this study, for the first time, the stabilities of reference genes in insect cell lines with CPT treatments were evaluated. Consistent with the previous results, the ribosomal proteins RPL13 and RPL3 were the most stable in expression and are most suitable as reference genes among the genes examined. GAPDH, on the other hand, was unstable in gene expression in Sf9 cells treated with camptothecin. Thus, RPL13 and RPL3 were recommended for normalization in Sf9 cells with CPT treatments.

Irradiation with ultraviolet light was recognized as another important factor to induce apoptosis via reactive oxygen species production and DNA lesions^45,46^. Apoptosis has been induced by ultraviolet in different insect cell lines, including S2, Sf9 and BM-N (*B. mori*)^11,47,48^. So far, Information on choices for reference genes in insects under UV irradiation is limited. RPS3, RPL13A and β-actin have been reported to be more stable as reference genes among seven analyzed genes in *Tribolium castaneum* (Herbst) (Coleoptera: Tenebrionidae) after UV irradiation^49^. In addition, β-actin and EF1α were confirmed to be the stable genes in brown citrus aphid, *Toxoptera citricida* (Kirkaldy) under UV irradiation stress^50^. In this study, for the first time, the expression stability of eight selected genes was evaluated in insect cells irradiated by ultraviolet. EF1α and β-1-TUB were found to be the most stable genes, whereas the ribosomal proteins and GAPDH were suggested to be the unstable. We speculate that some aspects of the cell lines *in vitro* could be different from the insect *in vivo*, so different results generated in different samples with ultraviolet treatment.

According to our results, the best stable genes for Sf9 cells under different conditions were varied. EF2 and EF1α were the best choices for Sf9 cells treated with azadirachtin, RPL13 and RPL3 for cells treated with camptothecin, EF1α and β-1-TUB for cells irradiated with ultraviolet. In addition, the stability of reference genes in Sf9 cells infected by *Heliothis virescens* ascovirus 3 h were assessed and 28 S was the stable one^51^. Hence, the expressions of reference genes are not static in samples with different conditions and it is necessary to verify the stabilities before RT-qPCR analysis. Furthermore, the stabilities of reference genes among Sf9 cells with same treatment were revealed based on the results of different calculation methods. For all the samples, the stability rankings of reference genes were similar according to all the analytical tools except BestKeeper. For example, EF2 and EF1α were assessed as the stable genes in all samples by Delta CT, geNorm, Normfinder and RefFinder. However, these two genes were regarded as unstable genes by BestKeeper. BestKeeper analyze the stability of reference genes just relying on the amplification efficiency of primers and Ct values^28^. However, other algorithms consider the pairwise variation of two reference genes as the important factor to determine the stability results. Thus, BestKeeper results differed from other algorithms. In addition, the pairwise variation value of geNorm is recommended as the judging index for optimal number selection of reference genes. In this study, all the pairwise variation values of samples were below 0.15, therefore, two reference genes should be chosen for RT-qPCR data normalization in Sf9 cells under apoptosis conditions. To our knowledge, the present study is the first report on stable evaluation of reference genes under apoptosis condition in Sf9 cells. Azadirachtin, camptothecin and UV light were considered as the extrinsic apoptosis stimuli and the expression stabilities of reference genes were varied in different situations. However, the expression stabilities of reference genes under apoptosis conditions induced by intrinsic signals still not revealed and need to be further exploration.

In the present study, eight candidate reference genes of Sf9 cells were selected and the primer specificity of each gene was identified. Meanwhile, the expression stability of eight reference genes was evaluated systematically by different algorithms and the optimal numbers used for qRT-PCR data normalization under different conditions were determined. Our results showed that the expression stability of different genes for Sf9 cells varied significantly under different conditions. Based on the variation, we recommend to use EF2 and EF1α as reference genes for Sf9 cells treated with azadirachtin; RPL13 and RPL3 for cells treated with camptothecin; and EF1α and β-1-TUB for cells irradiated with ultraviolet. The recommended reference genes shall result in more accurate estimation of RT-PCR data in future studies on apoptosis in Sf9 cells.

## Material and Methods

### Cell culture

Sf9 cells were maintained in 25 cm^2^ culture flasks with Grace’s insect medium (Gibco, USA) which supplemented with 10% fetal bovine serum (Gibco, USA), 0.33% yeast extract and 0.33% lactalbumin hydrolysate. The medium was changed every 2 days and the cells were subcultured until more than 80% coverage.

#### Treatments and samples collection

Sf9 cells were seed onto 6-well plates with the density of 1 × 10^5^−5 × 10^5^ cells/mL and incubated overnight at 28 °C. Cells were treated with 1 μg/mL azadirachtin (AZA) for 12, 24 and 36 h, respectively. The samples were collected by centrifugation of 8000 rpm for 5 min. For camptothecin (CPT) samples, the cells were treated with 1 μg/mL for 6, 12 and 24 h, respectively and collected as above. For ultraviolet (UV) samples, cells were irradiated after removing media with a 60 cm distance under a UV light, which has the power of 30 w for 5 min. Then cells were incubated in new medium for 6, 12 and 24 h, respectively. After that, the cells were collected as above.

### RNA extraction and quantitative reverse transcription

Total RNA was extracted by RNAiso plus (Takara, Japan) following the manufacturer’s instructions. Briefly, cells were homogenized into RNAiso plus reagent, and then incubated for 5 min at room temperature. Equal volume of chloroform (200 μL) was then added to the tube, and mixed fully with the lysate by vortexing for 15 s. The mixture was incubated for another 5 min at room temperature and centrifuged with 12000 rpm for 10 min at 4 °C. The supernatant of 500 μL was transferred to a new tube, and mixed with equal volume of isopropanol. After incubation for 10 min at room temperature, the mixture was then centrifuged under 12000 rpm for 10 min at 4 °C. The supernatant was removed and precipitate was washed with 75% ethanol. The precipitate was dried for 3 min at room temperature and dissolved with DEPC water. The purity and concentration of the RNA samples were analyzed on a NanoDrop^®^ spectrophotometer (Thermo Fisher, MA, USA).

The cDNA template for RT-qPCR was synthesized by a PrimeScript RT reagent Kit with gDNA Eraser (TaKaRa, Japan) following the operation manual. Potential genomic DNA contaminant was removed by gDNA Eraser. The first step for DNA digestion was performed in a 10 μL reaction system (1 μg total RNA, 2 μL 5 × gDNA Eraser Buffer and 1 μL gDNA Eraser) at 42 °C for 2 min. Then the reaction solution was mixed with another 10 μL solution which contained 4 μL RNase free water, 4 μL 5 × PrimeScript Buffer 2, 1 μL RT Primer Mix and 1 μL PrimeScript RT Enzyme Mix I, and incubated with the protocol of 42 °C for 15 min, 85 °C for 5 s. The cDNA was stored at −20 °C and later used as template for RT-qPCR.

### Primer design and qRT-PCR

Sequences of the seven selected genes were retrieved from Genbank in National Center for Biotechnology Information (NCBI) website. The accession numbers were actin (HQ008727.1), EF1α (U20139.1), GAPDH (KC262638.1), RPL3 (AY072287.1), RPL13 (AF400183.1), α-TUB (HQ008728.1) and β-1-TUB (AF548017.1). EF2 were not found in NCBI and were retrieved from transcriptomic data of Sf9 cells (SRA database accession number: SRR5892097)^1^. Primers of genes were designed by Primer Premier 5.0 (Premier, Canada). In order to establish a standard curve, RT-PCR was performed with LA *Taq* (Takara, Japan) and the PCR products of genes were purified by Universal DNA Purification Kit (TIANGEN, China). Then the purified PCR products were diluted as ten-fold and a series of concentration templates were formed and used for standard curves generation.

RT-qPCR experiments were fulfilled by iTaq^TM^ Universal SYBR^®^ Green Supermix (BIO-RAD, USA) on a CFX Connect^TM^ Real-Time System (BIO-RAD, USA). The reaction solution contained 5 μL iTaq^TM^ Universal SYBR^®^ Green Supermix, 0.5 μL of each primer, 0.5 μL cDNA and 3.5 μL sterile water and the reaction program was executed as follows: the denaturation step with 95 °C for 2 min, the amplification step with 40 cycles of 95 °C for 20 s, 60 °C for 15 s, and 72 °C for 15 s, and finally followed with the melting-curve step of 95 °C for 10 s and 65 °C for 5 s.

### Data analysis

Each treatment was carried out with three replicates and the Ct values of reference genes in different samples were collected. Furthermore, expression stability of eight reference genes was evaluated by Delta Ct method, BestKeeper, geNorm, NormFinder and RefFinder (http://www.leonxie.com/referencegene.php). Among them, the stability of reference genes evaluated by Delta CT method depends on the relative expression of pairwise genes within each sample^52^. Besides, BestKeeper was considered as another algorithm common used for stability assessment of reference genes. Simultaneously, the amplification efficiency of primers and the Ct-values of reference genes obtained from RT-qPCR were the crucial factors for this algorithm^53^. In addition to examine the stability of candidate reference genes, geNorm was also used to determine the appropriate number of reference genes for the standardization of RT-qPCR results. In this algorithm, the stability value (M) obtained from geNorm and pair-wise variation value (V) were the determinants. M assess the reference genes’ stability and V determine the optimal number, in which V < 0.15 was the criterion for better normalization^54^. Meanwhile, the optimal normalization reference genes for RT-qPCR analyzed by NormFinder were obtained through estimating the expression variation^55^. Furthermore, RefFinder was adapted for an intelligent ranking of selected genes based on their stability in gene expression levels.
